# Antimicrobial Susceptibility Patterns of *Shigella* Species among Children under Five Years of Age with Diarrhea in Selected Health Centers, Addis Ababa, Ethiopia

**DOI:** 10.1155/2023/5379881

**Published:** 2023-08-10

**Authors:** Basha Ayele, Zeleke Mekonnen, Tesfaye Sisay Tessema, Etsehiwot Adamu, Estifanos Tsige, Getenet Beyene

**Affiliations:** ^1^Department of Medical Laboratory Science, College of Health Science and Medicine, Dilla University, P.O. Box 419, Dilla, Ethiopia; ^2^School of Medical Laboratory Sciences, Institution of Health Sciences, Jimma University, Jimma, Ethiopia; ^3^Institution of Biotechnology, Addis Ababa University, Addis Ababa, Ethiopia; ^4^Ethiopian Public Health Institute, Addis Ababa, Ethiopia

## Abstract

**Background:**

*Shigella* and parasitic infections are common public health problems throughout the world. Shigellosis is an acute gastroenteritis infection and one of Ethiopia's most common causes of morbidity and mortality, especially in children under five. High resistance rates to commonly used antibiotic agents have been documented in different locations in Ethiopia.

**Objective:**

This study aimed to characterize the antimicrobial features of the *Shigella* species isolated from children under five years of age with acute diarrhea in Addis Ababa, Ethiopia.

**Methods:**

Using a cross-sectional study, freshly passed fecal specimens were collected for intestinal parasite and bacterial isolation. Fecal samples for bacterial identification were placed immediately in Cary–Blair media and transported to the Ethiopian Public Health Institution (EPHI) laboratory. Antimicrobial susceptibility testing (AMST) was conducted using the disk diffusion method. Data were described using descriptive statistical tools. The association of independent and dependent variables was evaluated with logistic regression. A *P* value ≤0.05 was considered statistically significant.

**Results:**

The prevalence of intestinal parasites was 8.2% with seven different species. Among the 534 stool-cultured specimens, 47 (8.8%) were positive for *Shigella* species. Antimicrobial susceptibility testing (AMST) showed that 100%, 93.6%, 80.9%, 72.3%, and 57.5% were susceptible to norfloxacin, nalidixic acid, ciprofloxacin, gentamicin, and cefoxitin, respectively. However, 100% of the isolates were resistant to amoxicillin and erythromycin. More than 50% of the isolates were resistant to three and above antibiotics, while none of them were susceptible to all the antibiotics tested. All risk factors assessed did not show a statistically significant association with *Shigella* infection.

**Conclusion:**

The high levels of antibiotic resistance observed among the commonly prescribed antibiotics are alarming. The emerging resistance to ciprofloxacin and nalidixic acid signals a severe public health threat in the management of shigellosis. Raising awareness about resistance and educating health professionals, policymakers, and the public can help improve the quality of patient care and rational antibiotic use.

## 1. Introduction

The burden of diarrheal disease is highest in developing countries associated with parasite and *Shigella* infection where there is poor sanitation, inadequate hygiene, unsafe drinking water, and poorer overall health and nutritional status [1]. Shigellosis is caused by the ingestion of bacteria of the genus *Shigella*. It is gram-negative enteropathogenic bacteria responsible for human diarrhea and dysentery. There are four species of *Shigella* based on serological and biochemical characteristics: *Shigella dysenteriae (S. dysenteriae), S. flexneri, S. boydii,* and *S. sonnei* [2]. Species or serogroups are further subdivided into serotypes based on “the O antigen of a lipopolysaccharide outer cell wall membrane.” It is a germ that is extremely infectious; only 10 bacilli are required to cause infection [3]. Serogroup A (*S. dysenteriae*) has 15 serotypes and 2 provisional serotypes [4]. Serogroup B (*S. flexneri*) has 6 serotypes and 16 subserotypes. Serogroup C (*S. boydii*) has 20 serotypes, and serogroup D (*S. sonnei*) has only 1 serotype [5]. Morbidity and mortality result from inadequate hand washing after defecation or nappy changing or from person-to-person directly via the fecal-oral route [6]. Serogroup D (*S. sonnei*) is the most physiologically distinct and can be distinguished by key biochemical and genetic characteristics, including lactose fermentation and differences in the utilization of a range of substrates. *Shigella sonnei* (*S. sonnei*) can be divided into five biotypes (a, b, e, f, and g) based on biochemical properties [4].

Disease usually begins with fever, fatigue, anorexia, and malaise. Some patients have mild watery diarrhea, whereas others experience severe dysentery with systemic complications such as electrolyte imbalance, seizures, and hemolytic uremic syndrome [7]. Shigellosis is the most common cause of epidemic dysentery and is found all over the world. In developing countries, it is the leading cause of infant diarrhea and mortality [2]. The burden of *Shigella* in children under five years of age was variable from region to region. The prevalence of shigellosis in Ethiopia was reported to be high in children [8]. *Shigella flexneri* (*S*. *flexneri*) is dominant in Africa and Asia, although *S. sonnei*, the most dominant species in South America, was the predominant isolate in one study in Ethiopia [9]. This may give clues to the scientific world about the migration and movement of strains from one region to another. In Ethiopia, it is difficult to evaluate the burden of *Shigella* infection because the diagnosis is on clinical grounds and there is a lack of coordinated epidemiological surveillance systems. In addition, underreporting of cases and the prevalence of other diarrhea illnesses regarded as high priorities may have obscured the shigellosis issue. Since *Shigella* species are not frequently grown and their antibiotic resistance cannot be tested in Addis Ababa health centers, more research is needed to address the true status of their antibiotic resistance. Antimicrobial resistance (AMR) patterns vary from region to region and even within a single region [4]. The majority of diarrheal illnesses in Ethiopia are treated clinically without laboratory confirmation, mainly in health centers.

Increasing prevalence of multidrug resistance (MDR) to *Shigella* species is a serious threat, especially in Ethiopia with health and nutritional problems. There was identified MDR among several serotypes of *Shigella* species isolated from acute diarrheal patients [10]. Irrespective of the serogroup/serotype, most of the strains carried similar genes encoding resistance to specific antimicrobials. This emergency of drug resistance calls for the rational use of effective drugs and underscores the need for alternative drugs to treat infections caused by resistant strains. Resistance to commonly used antibiotics is high; therefore, it is important to look at the susceptibility pattern before starting treatment. The occurrences of invasive bacterial infections from numerous sites were presented, revealing not only the presence of *Shigella* species infection but also significant continent-wide variation in estimated disease prevalence and antimicrobial susceptibility patterns. Therefore, this study aimed to determine the prevalence and antimicrobial sensitivity pattern of *Shigella* species infections among under-five children in selected health centers in Addis Ababa, Ethiopia.

## 2. Methods

### 2.1. Study Sites

The investigation was carried out in Addis Ababa, Ethiopia, in the outpatient pediatric departments of the chosen medical facilities (Wereda 1 health center, Wereda 2 health center, Arada health center, and Shegole health center) from June 2021 to April 2022. Addis Ababa is the capital of Ethiopia and one of the largest cities on the African continent, with an altitude of 2,355 meters above sea level and an annual temperature ranging from 18°C to 25°C. Addis Ababa has a total population of 3,602,000 as of the 2019 projection based on the 2007 census, consisting of 1,134,150 children under the age of five [11].

### 2.2. Study Design

A laboratory-based cross-sectional study design was conducted in order to obtain stool specimens and related information from children with acute diarrhea (within 7 days) in Addis Ababa, Ethiopia.

### 2.3. Study Population and the Sampling Process

First, the sampling technique was carried out by listing the subcities of Addis Ababa. Second, by the simple random method, three subcities (Gulele, Kolfe Keranio, Arada, and Nefassilk Lafto) were selected. Then, four health centers were also selected by the simple random method. 534 under-five children with diarrhea who met the inclusion criteria during the study period were chosen using the systemic sample method based on population allocation of the four health centers utilizing the case interval. By using a lottery, the first case of the interval was chosen. The study included all under five-year-old children with acute diarrheal disease (144 children from Wereda 1 health center and 390 children from the other three health centers (130 children from each)). Children whose parents/guardians were not voluntary to participate, newborn, inpatients, and those who had persistent diarrhea and had taken antimicrobial treatment 1 week prior to and at the time of data collection were excluded from the study.

### 2.4. Data and Sample Collection

The study was conducted in four public health facilities. Data related to the sociodemographic profile of the children and associated factors were collected using a pretested questionnaire adapted from the relevant literature; data were collected by face-to-face interviews [12–14]. Data were collected by four clinical nurses and four medical laboratories. After the physicians established that the children had acute diarrhea, data collectors interviewed the parents or guardians on behalf of the children at the health facility. The age of the children information was verified from birth certificates. Prior to collecting data, parents and guardians were fully informed of the study's objectives and the advantages of participation. Participation was on voluntarily basis, and they were informed that it is within their right to withdraw from the study at any time during the course of the study. Diarrhea-suspected children that were in the pediatrics department during the first day of the study period and registered from the case interval were the first subject of the study. After proper instruction using their identification card (ID), each caregiver was asked to bring the stool specimen from their children in clean, dry, leak-proof disposable stool cups. Approximately, 1 g of stool was immediately transferred into the Cary–Blair transporting medium (Oxoid Ltd., Basingstoke, UK), labeled, and transported within 1-2 hours of collection in an ice-packed cold box (4°C) to the Ethiopian Public Health Institute (EPHI) of Bacteriology and Mycology Laboratory for bacterial identification.

### 2.5. Culture and Identification

Isolation and characterization of *Shigella* species were performed based on the practical guideline and handbook of clinical microbiology procedures [15, 16]. In order to enrich the bacteria, a mixture of a feces sample (1 mL) was added to a tube containing 9 mL of Selenite F broth (Oxoid Ltd., UK) and incubated at 37°C for 24 hours. Then, an inoculum was transferred from Selenite F broth into MacConkey agar (MAC) and xylose lysine deoxycholate (XLD) agar plates. *Shigella* growth was distinguished from other lactose-negative suspected colonies on MAC by their distinctive colony morphology on XLD agar after subculture and overnight incubation at 37°C. Suspected *Shigella* colonies' further identification was performed biochemically using oxidase, Kligler iron agar (KIA), urea, indole, citrate, SIM media, and lysine iron agar (lysine decarboxylase (LDC)) tests. After incubation for 18–24 h at 37°C, the test media were read for characteristic *Shigella* species reactions.

### 2.6. Intestinal Parasite Identification

After interviewing, respondents were asked to give a fresh stool specimen in a sterile, clean, wide-mouthed plastic container by using a clean wooden applicator stick, which was transported to the microbiology laboratory for analysis. Two stool samples were collected; one stool sample was immediately emulsified using saline (0.85% NaCl) for parasitological examination of trophozoite, cyst, larva, and ova stages by direct wet mount preparation in normal saline and iodine solution, and the second stool was used for parasites identification using formol-ether concentration sedimentation techniques.

### 2.7. Antimicrobial Susceptibility Testing

According to the standard operating procedure (SOP) adopted from the Clinical Laboratory Standards Institute (CLSI) [17], the antimicrobial susceptibility test (AMST) was carried out using the Kirby–Bauer single disk diffusion method on Muller–Hinton agar (MHA) (Oxoid, Hampshire, UK) plates. To produce a homogeneous suspension according to 0.5 McFarland densitometer standards, three to five pure colonies were selected using a sterile loop and mixed with sterile normal saline (0.85 percent NaCl). Using a sterile cotton swab, the suspension was evenly distributed onto an MHA plate. For 3–5 minutes, the plate was dried at room temperature. Antimicrobial disks used in the study were ampicillin (10 *μ*g), chloramphenicol (30 *μ*g), cotrimoxazole (25 *μ*g), gentamycin (10 *μ*g), amoxicillin (10 *μ*g), doxycycline (30 *μ*g), tetracycline (30 *μ*g), erythromycin (15 *μ*g), cefoxitin (10 *μ*g), ciprofloxacin (5 *μ*g), nalidixic acid (30 *μ*g), and norfloxacin (10 *μ*g). Based on the zone of inhibition, the disks were measured using a ruler to the nearest whole millimeters after the plates (3-4 antibiotic disks on a 100 mm plate) were incubated at 37°C for 24 hours. By comparing the diameter of the standard with the current inhibition zone, the result was classified as susceptible or sensitive (S), intermediate (I), and resistant (R). The technique was closely adhering to the SOP for the preparation and standardization in order to standardize the inoculum density for a susceptibility test. The term “multiple drug resistance” (MDR) was defined as the resistance of bacterial isolates to two and more antibiotics in more than two antimicrobial categories tested [18–20].

### 2.8. Data Quality Control

Training was given to data collectors. The English version of the questionnaire was translated to local language (Amharic). Control strains were used during the microbiological investigation of AMST and checking the efficiency of culture media. The standard operating procedure (SOP) and CLSI were used throughout the procedures. In addition, sterility of the culture media was checked frequently by incubating the prepared culture media at 37°C overnight and checked for growth. American Type Culture Collection (ATCC) reference strains were used to test the performance of each culture medium and antimicrobial disks before use. To test the quality of MAC agar, ATCC was used to inoculate the MAC agar media with *Proteus mirabilis* (*P. mirabilis*) ATCC®35659, *Escherichia coli* (*E. coli*) ATCC®25922, and *Enterococcus faecalis* (*E. faecalis*) ATCC®29212. To assess the quality of the XLD agar medium, *P. mirabilis* ATCC®35659, *E. coli* ATCC®25922, and *Staphylococcus aureus* (*S. aureus*) ATCC®25923 were used. Before using an antimicrobial disk, MHA was tested for effectiveness on an agar plate using the strains of *E. faecalis* ATCC® 29212, *S. aureus* ATCC® 25923, and *E. coli* ATCC® 25922 growth. *Escherichia coli (E. coli)* ATCC®25922, *S. aureus* ATCC®25923, and *Pseudomonas aeruginosa (P. aeruginosa)* ATCC®27853 were included as reference strains for AMST. The temperature used during storage of the disks, materials, and regents was based on the manufacturer's instruction.

### 2.9. Data Analysis

EpiData version 3.1 (EpiData Association, Odense, Denmark) was used to input the data, and STATA version 14 software program (Stata Corporation, Lakeway Drive, College Station, Texas) was used to export it for analysis. The frequency, percent, mean, and standard deviation of the data were used to explain the data using descriptive statistical techniques. The outcome variables' predictors were found using independent variables such as sociodemographic and clinical characteristics. The rate of *Shigella* isolates was examined using descriptive statistics. To determine the adjusted odd ratio (AOR) and *P* value, logistic regression was used to assess the relationship between the independent and dependent factors in the binary outcome statistics. In the logistic regression analysis with a 95% confidence interval (CI), *P* ≤ 0.05 was considered statistically significant.

## 3. Results

### 3.1. Sociodemographic Characteristics

Out of the 534 study participants, 167/534 (31.3%) were females. The participants in the study ranged in age from 6 months to 4.11 years (mean age: 2.14 years ± 1.04). Of the age categories, 18/534 (3.4%) of them were infants and 179/534(33.5%) were between 2 and 2.11 years old. About 12.7% caregivers or parents of the children were illiterate, while 5.6% of parents were first degree and above educational holders. Twelve of the children lived with a single parent. Among the parents of the children, 71.2% were housewife. Overall, about 50.9%, 60.1%, and 91.8% of the participants had four families, 3000–4000EB monthly income, and one child in the household, respectively (Table 1).

### 3.2. Clinical Characteristics

There was no history of antibiotics use by children in the past two weeks prior to and at the time of data collection. The most common clinical complaints recorded were mixed illness (diarrhea vomiting, abdominal pain, and fever) (40.3%), followed by watery diarrhea (36.9%) (Table 1).

### 3.3. Prevalence of *Shigella* Species and Associated Risk Factors

As shown in Table 1, 47 (8.8%) of the stool specimens tested positive for *Shigella* species. *Shigella* species isolated from male (*n* = 34; 6.4%) had a higher frequency than female (*n* = 13; 2.4%) children. The age range between 1- and 2.11-year-old had the largest percentage of infections (5.4%), whereas no pathogen was isolated from less than 1-year-old. Children with two days of diarrhea, four family sizes, occupational (housewife), monthly income (3001–4000EB), and one child in the household were more likely to be exposed to *Shigella* infection although the difference was not statistically significant (*P* > 0.05). All the study participants visited the health center 1–5 days after the onset of diarrhea. In general, all risk factors assessed did not show a statistically significant association with the presence of *Shigella* infection.

### 3.4. Prevalence and Types of Intestinal Parasites

The overall prevalence of intestinal parasites was 8.2% with seven different species. *Entamoeba histolytica* was the leading parasite isolated (3.0%), followed by *Ascaris lumbricoides* (1.9%), and least parasites isolated were *Giardia lambelia* (0.4%). No mixed parasite infection was isolated from the study participants (Table 2).

### 3.5. Antimicrobial Susceptibility Pattern of the Isolated *Shigella* Species

**Figure 1:**
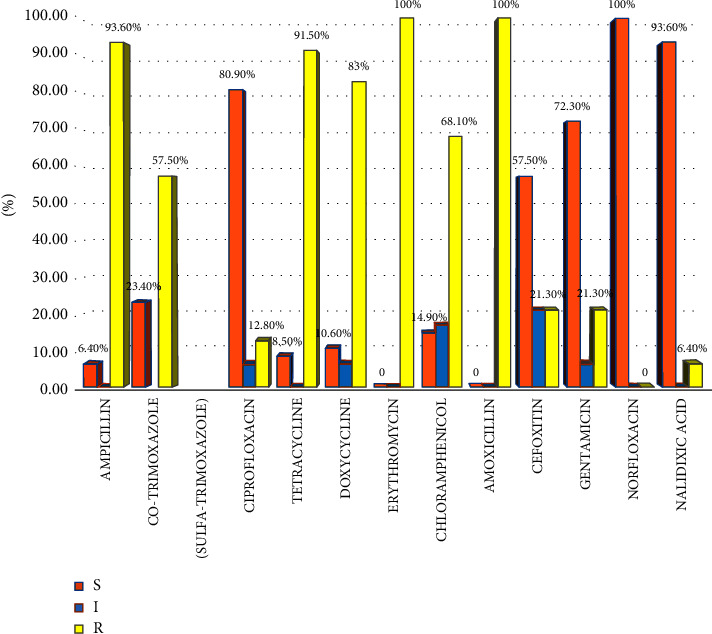
Antimicrobial resistance pattern of the *Shigella* species isolated from stool cultures among under-five diarrheic children. S = sensitive; I = intermediate; R = resistant.

The antimicrobial susceptibility patterns of *Shigella* isolates were tested against 12 selected antibiotics, and generally, more than half of the isolates showed resistance to tested antibiotics. With respect to susceptibility, 100%, 93.6%, 80.9%, 72.3%, and 57.5% of the isolates were susceptible to norfloxacin, nalidixic acid, ciprofloxacin, gentamicin, and cefoxitin, respectively. However, all isolates were 100% resistant to amoxicillin and erythromycin (Figure 1).

Out of the 47 *Shigella* isolates, 40 (85.1%) were identified as MDR. More than 50% of the isolates were resistant to three and more antibiotics, while none of them were susceptible to all the antibiotics tested. High levels of resistances were observed for erythromycin, amoxicillin, and ampicillin. Nearly, 55.3% of the *Shigella* isolates have developed resistance to four antibiotics, followed by 36.2% resistant to five antibiotics, 12.8% to six antibiotics, and 4.3% of isolates were resistant to seven antibiotics (Table 3).

## 4. Discussion

As one of the most prevalent causes of epidemic dysentery, shigellosis is virtually universal. It is the main contributor to newborn mortality and diarrhea, primarily in developing nations [2]. In Ethiopia, *Shigella* species are not regularly cultured for routine patient diagnosis, and their antibiotic resistance cannot be verified. Antimicrobial resistance (AMR) patterns vary from region to region and even within a single region [4].

In the current investigation, 47 (8.8%) children under the age of five had positive stool cultures for *Shigella* species. *Shigella* poses a serious threat to the public's health because only a small number of cells (10–100 bacilli) are required to generate bacillary dysentery, which can result in shigellosis [3]. Comparable to this finding, the reported isolation rates in the previous research conducted in Ethiopia were 8.3% in Hossana [18], 9.1% in Addis Ababa [21], 9.5% in Adama [22], and 6.9% in Mekelle [23]. The outcome is, however, less than those from Iran (12.6%) [24] and Kenya ((14.8%) [25] and (23.6%)) [26]. The possible explanation for this variance could be attributed to variations in the sample, regional variation, and socioeconomic situations. In addition, the difference in prevalence may be caused by an improved standard of living, increased understanding of sanitation and hygiene, and the use of preventative measures to control infectious diseases. Therefore, greater community awareness, particularly among mothers, about personal and environmental hygiene directly affects the prevalence of *Shigella* species in children. This study showed a higher rate of *Shigella* species isolation compared to reports from Juba, South Sudan (5.2%) [27], Arba Minch, Ethiopia (4.8%) [12], Robe and Goba, Ethiopia (4.3%) [28], Adigrat, Ethiopia (3.7%) [29], Ambo, Ethiopia (2.5%) [14], Brazil (2.2%) [30], Debre Markos, Ethiopia (2.3%) [31], Nepal (2.1%) [32], and Jimma, Ethiopia (1.1%) [33]. This might be because of variations in methodology, study participant types, sample sizes, and geographic locations. The prevalence of *Shigella* species reports varied in different regions and time [7]. Children under the age of five are naturally taking contaminated soils, food, and water into their mouth and may acquire disease, causing microbes including *Shigella* species [28]. According to a review study conducted in Ethiopia [8], the pooled prevalence of shigellosis in children was 7.0%, whereas it was 2.2% in the adult population. This reveals that newborns and young children are more susceptible to diarrheal morbidity due to *Shigella* than adults.

Similar to the current study, earlier research conducted on children under the age of five found no statistically significant link between *Shigella* infection and sociodemographic characteristics [12, 18]. In the current study, infants were not associated with *Shigella* infection. The most plausible explanation for this outcome is that the mother's immunity, which can shield the infant from infection for about six months, or that the parents provide their child adequate medical care. In the present investigation, children aged 1–2.11 years had a greater prevalence of *Shigella* species though this was not significant. This result was consistent with multiple investigations [12, 18, 28] which showed that children with a lower immunological state and those whose parents had lower educational backgrounds are more prone to *Shigella* infection. This study reported a more isolation rate of *Shigella* species among those with low educational background in connection to educational status and the frequency of isolation (1–8 grade). This result is analogous with the past research in south Ethiopia [34]. To control other factors that cause this disease and to raise community awareness of the mechanisms for managing infectious diarrhea, education is essential. Poor environmental sanitation, malnutrition, inadequate water supply, poverty, and limited education are the major factors implicated in the occurrence, spread, and severity of diarrheal disease [35].

One to five days following the commencement of diarrhea, all study participants went to the health centers. In this investigation, the most common symptoms of *Shigella* cases with a positive culture were fever, vomiting, and abdominal pain. This is caused by the bacteria's capacity to enter and reproduce in the cells lining the colon and rectum. Similar results have been reported from Asia [36]. In northern Ethiopia, more *Shigella* isolates were found in bloody and mixed (blood and mucus) samples [23]. In the current study, patients with watery diarrhea and mixed (mucus and blood) samples were more susceptible to *Shigella* species. This contrary outcome may be due to variations in the unidentified strains connected to the patient or to the fact that more patients with watery diarrhea than bloody diarrhea participated in this investigation.

In the current study, *Entamoeba histolytica* was the leading parasite isolated (3.0%), followed by *Ascaris lumbricoides* (1.9%) and least parasites isolated were *Giardia lamblia* (0.4%). A similar finding has been reported in the previous study conducted in Addis Ababa [37]. The predominant intestinal parasite reported in Aksum, Ethiopia (5%) [38], Iran (3.7%) [39], and Saudi Arabia (9%) [40] was *Giardia lamblia*; however, *Ascaris lumbricoides* was reported in Dilla, Ethiopia (11.4%) [41]. The high prevalence of those parasites might be due to low personal hygiene practice with easy transmission of the parasite which is usually found in unsafe food, water, soil, or contaminated surface.

In developing nations like Ethiopia, where infectious illnesses are prevalent, AMR is a major global public health concern. *Shigella* species were tested in this study against 12 antibiotics, and the results showed a variety of sensitivity and resistance patterns. In the current and earlier research in Ethiopia, both patterns have shown differences. By 2050, a person will die every three seconds if antibiotic resistance is not addressed [42]. In the current investigation, *Shigella* species showed a high frequency of isolates resistant to antimicrobial drugs, although some of the isolates were also sensitive to norfloxacin, nalidixic acid, ciprofloxacin, cefoxitin, and gentamicin. This may be linked to the widespread usage of basic antibiotics and their simplicity of availability. In Ethiopia, it is simple for anyone to visit a pharmacy or a drug vendor and get the most common antibiotics without a prescription [14, 43]. The high resistance rates seen could be due to this and other malpractices such as the use of lower dosages, improper care of antibiotics for instance selling antibiotics in open markets in rural areas of the country, and low efficacy of imported drugs [44]. The resistance patterns of *Shigella* against antimicrobial drugs in the present study range from 100% (for amoxicillin and erythromycin) to 6.4% (for nalidixic acid); however, norfloxacin was 100% susceptible to all isolates, which is in agreement with the previous report from Ethiopia [7]. The three most crucial medications used to treat shigellosis in Ethiopia are amoxicillin, cotrimoxazole, and ciprofloxacin. In the treatment of shigellosis, the emergence of resistance to ciprofloxacin and nalidixic acid raises serious public health concerns. The Clinical and Laboratory Standards Institute (CLSI) on performance standards for AMST stated that the result of ampicillin can be used to predict the result for amoxicillin and tetracycline for doxycycline regardless of the finding [17]. Ampicillin and doxycycline resistance patterns in the current investigation were 93.6% and 83%, respectively. The resistance pattern of amoxicillin (100%) in this study was similar to that of studies carried out in other regions of Ethiopia [28, 45, 46] and also showed comparable resistance to tetracycline (91.5%) with earlier studies in Bahir Dar (93.8%) [47] and Gonder (89.7%) [48]. According to Tosisa et al. [14], amoxicillin resistance was present in 50% of *Shigella* species in Ambo, Ethiopia, which is contrary to the findings of this study. This can be because the study site sources and laboratory standards employed differed. The current data showed complete resistance against erythromycin, which is consistent with a research conducted in Debre Markos, Ethiopia [49]. The accessibility and prolonged use of the drug could be the reasons for such levels of resistance. *Shigella* isolates create R plasmids, which can confer several antibiotic resistances and contain numerous resistance genes [50]. Similar analyses of the genes responsible for antibiotic resistance in *Shigella* isolates from southeastern Africa that caused diarrhea in children under the age of five revealed the presence of oxa-1-like-lactamases for ampicillin, dfrA1 genes for trimethoprim-sulfamethoxazole/cotrimoxazole, tetB genes for tetracycline, and Chl acetyltrans [51]. Limiting the findings to *Shigella* species made identifying other common causes of diarrhea more difficult. This study included only children with acute diarrhea and was limited to public health facilities. Despite these limitations, the findings of this study may be useful in informing clinicians, public health officials, and researchers about *Shigella*-associated diarrhea, its contributing causes, and the antibiotic susceptibility pattern.

## 5. Conclusion

In conclusion, 8.8% of the children under the age of five tested positive for *Shigella* species in the study, which involved all study participants visiting the health facility between one and five days after the onset of diarrhea. The high prevalence of *Shigella* species with antibiotic resistance, combined with MDR among commonly prescribed antibiotics such as ampicillin, cotrimoxazole, tetracycline, erythromycin, and chloramphenicol, necessitates immediate strengthening of the monitoring mechanism on the prudent use of antibiotics as well as a broad-based behavioral change. The emerging resistance to ciprofloxacin and nalidixic acid signals a serious public health threat in the management of shigellosis. Raising awareness about resistance and educating health professionals, policymakers, and the public can help improve the quality of patient care and the rational antibiotic use.

## Figures and Tables

**Table 1:** Prevalence of *Shigella* isolates and associated factors among under-five diarrheic children with acute diarrhea in the selected health centers, Addis Ababa (June 2021–April 2022) (*n* = 534).

Variables	Patient frequency (%^a^)	*Shigella* isolates (%^b^)	AOR (95% CI lower-upper)	*P* value
Sex				
Male	367 (68.7)	34 (6.4)	1.2 (0.62–2.39)	SNS
Female	167 (31.3)	13 (2.4)	1	
Age				
Less than 1 year	18 (3.4)	—	—	SNS
1 and less than 2 years	158 (29.6)	15 (2.8)	1.4 (0.59–4.01)	
2 and less than 3 years	179 (33.5)	14 (2.6)	1.3 (0.48–3.25)	
3 and less than 4 years	119 (22.3)	11 (2.1)	1.3 (0.47–3.53)	
4 and less than 5 years	60 (11.2)	7 (1.3)	1	
Educational status of the caregiver				
Illiterate	68 (12.7)	9 (1.7)	1	SNS
1–8 grade	139 (26.0)	14 (2.6)	1.4 (0.55–3.49)	
9–12 grade	184 (34.5)	11 (2.1)	1.2 (0.91–6.46)	
Diploma	113 (21.2)	10 (1.9)	1.1 (0.58–5.64)	
First degree and above	30 (5.6)	3 (0.6)	0.7 (0.32–9.85)	
Occupational status of the caregiver				
House wife	380 (71.1)	32 (6.0)	1	SNS
Governmental	43 (8.0)	3 (0.6)	0.6 (0.27–1.66)	
NGO	11 (2.1)	2 (0.4)	0.6 (0.07–2.15)	
Self-employee	65 (12.2)	7 (1.3)	0.7 (0.24–3.99)	
Private	35 (6.6)	3 (0.6)	0.6 (0.27–1.66)	
Monthly income				
<1000EB	1 (0.2)	—	—	SNS
1000−2000EB	19 (3.6)	1 (0.2)	0.6 (0.41–21.92)	
2001−3000EB	123 (23.0)	12 (2.2)	0.9 (0.46–2.15)	
3001−4000EB	321 (60.1)	29 (5.4)	1	
>4000EB	70 (13.1)	5 (0.9)	0.7 (0.31–3.97)	
Family size				
Two	12 (2.3)	1 (0.2)	0.6 (0.01–3.88)	SNS
Three	164 (30.7)	16 (3.0)	0.9 (0.08–6.4)	
Four	272 (50.9)	24 (4.5)	1	
Five	83 (15.5)	5 (0.9)	0.7 (0.12–11.94)	
More than five	3 (0.6)	1 (0.2)	0.6 (0.01–3.88)	
Number of children in a household				
One	490 (91.8)	44 (8.2)	1	SNS
Two	43 (8.0)	2 (0.4)	0.5 (0.04–8.81)	
Three	1 (0.2)	1 (0.2)	—	
Illness				
Diarrhea	132 (24.7)	14 (2.6)	0.9 (0.05–15.31)	SNS
Diarrhea and vomiting	82 (15.3)	8 (1.5)	0.8 (0.02–2.62)	
Diarrhea and abdominal pain	71 (13.3)	4 (0.7)	0.7 (0.08–6.13)	
Diarrhea and fever	34 (6.4)	2 (0.4)	0.6 (0.07–37.90)	
Mixed illness	215 (40.3)	19 (3.6)	1	
Duration of diarrhea				
One day	106 (19.8)	9 (1.7)	1.2 (0.30–6.17)	SNS
Two days	235 (44.0)	26 (4.9)	1.4 (0.46–4.24)	
Three days	145 (27.2)	9 (1.7)	1.2 (0.30–6.17)	
More than three days	48 (8.9)	3 (0.6)	1	
Duration of fever				
None	285 (53.4)	26 (4.9)	1.4 (0.51–21.24)	SNS
One day	33 (6.2)	2 (0.4)	0.8 (0.11–2.56)	
Two days	129 (24.2)	14 (2.6)	1	
Three days	68 (12.7)	3 (0.6)	0.8 (0.14–5.42)	
Four days	14 (2.6)	2 (0.4)	0.8 (0.11–2.56)	
More than four days	5 (0.9)	—	—	
Consistence of stool				
Watery	197 (36.9)	17 (3.2)	1.1 (0.45–2.44)	SNS
Mucoid	84 (15.7)	7 (1.3)	0.9 (0.31–2.25)	
Bloody	61 (11.4)	9 (1.7)	0.9 (0.35–2.33)	
Mixed (blood and mucus)	191 (35.8)	14 (2.6)	1	
Loose	1 (0.2)	—	—	

1%^a^, among total participants (*n* = 534); %^b^, *Shigella* positive within each row category; AOR, adjusted odd ratio; CI, confidence interval; EB, Ethiopian birr; —, no report; SNS, statistically nonsignificant association.

**Table 2:** Prevalence of intestinal parasites isolated from stool specimens of under-five children in Addis Ababa, Ethiopia (June 2021–April 2022) (*n* = 534).

Types of parasites	Frequency	Percentage (%)
*Entamoeba histolytica*	16	3.0
*Ascaris lumbricoides*	10	1.9
*Enterobius vermicularis*	7	1.3
*Trichuris trichiura*	5	0.9
*Hookworm*	4	0.7
*Giardia lamblia*	2	0.4
Total	44	8.2

**Table 3:** Multidrug resistance pattern of *Shigella* isolates from under-five children with acute diarrhea in the selected health centers of Addis Ababa (June 2021–April 2022) (*n* = 47).

Number of antimicrobial resistance	Antibiotics	Frequency (%)
*R*_1_	ERY	47 (100)
*R*_2_	ERY, AMP	44 (93.6)
*R*_3_	ERY, AMP, TET	40 (85.1)
*R*_4_	ERY, AMP, TET, CHL	26 (55.3)
*R*_5_	ERY, AMP, TET, CHL, COT	17 (36.2)
*R*_6_	ERY, AMP, TET, CHL, COT, FOX	6 (12.8)
*R*_7_	ERY, AMP, TET, CHL, COT, FOX, GEN	2 (4.3)

ERY: erythromycin; AMP: ampicillin; TET: tetracycline; CHL: chloramphenicol; COT: cotrimoxazole; FOX: cefoxitin; GEN: gentamicin. *R*_1_, *R*_2_, *R*_3_, *R*_4_, *R*_5_, *R*_6_, and *R*_7_ = resistant to one, two, three, four, five, six, and seven antibiotics, respectively.

## Data Availability

The datasets supporting the conclusions of this article are included in the article.

## Abbreviations

AMR: Antimicrobial resistance
AMST: Antimicrobial susceptibility testing
AOR: Adjusted odd ratio
ATCC: American Type Culture Collection
CI: Confidence interval
CLSI: Clinical Laboratory Standards Institute
EPHI: Ethiopian Public Health Institute
ID: Identification card
LDC: Lysine decarboxylase
MAC: MacConkey agar
MDR: Multidrug resistance
SOP: Standard operating procedure
XLD: Xylose lysine deoxycholate.
